# Crystallographic Texture in Ceramics and Metals

**DOI:** 10.6028/jres.106.057

**Published:** 2001-12-01

**Authors:** Mark D. Vaudin

**Affiliations:** National Institute of Standards and Technology, Gaithersburg, MD 20899-8524

**Keywords:** bulk and thin film samples, ceramics, crystallographic texture, metals, powder x-ray diffractometer

## Abstract

Preferred crystallographic orientation, or texture, occurs almost universally, both in natural and man-made systems. Many components and devices in electronic and magnetic systems are fabricated from materials that have crystallographic texture. With the rapidly increasing use of thin film technology, where sharp axisymmetric crystallographic texture normal to the film plane is frequently observed, the occurrence and impact of texture are rising. Thin film applications in which the texture of the material plays a key role in determining properties and performance are broad: complex oxides in random access memory devices, ZnO thin film resonators for cell phone applications, metallic alloys in magnetic recording media, and Al and Cu interconnects in integrated circuits are but a few examples. Texture is established during the synthesis or post-synthesis heat treatment of a material and thus has a strong dependence upon processing history. Accurate measurement of texture is not simple and a variety of tools and approaches are being actively employed in texture studies. X-ray, neutron and electron diffraction based techniques are practiced around the world at varying levels of complexity with regard to equipment and analysis methods. Despite the well-documented existence of these varied approaches, many reported texture measurements on electronic materials are based solely on the relative intensities of conventional *θ*-2*θ* x-ray diffraction peaks, which typically yield inaccurate results. NIST has developed quantitative texture measurement techniques that employ equipment commonly available in most industrial and academic settings. A number of examples of texture measurement in ceramic and metal systems will be presented, taken from the historical development and application of these techniques at NIST over the past 7 years.

## 1. Introduction

**Fig. 1:**
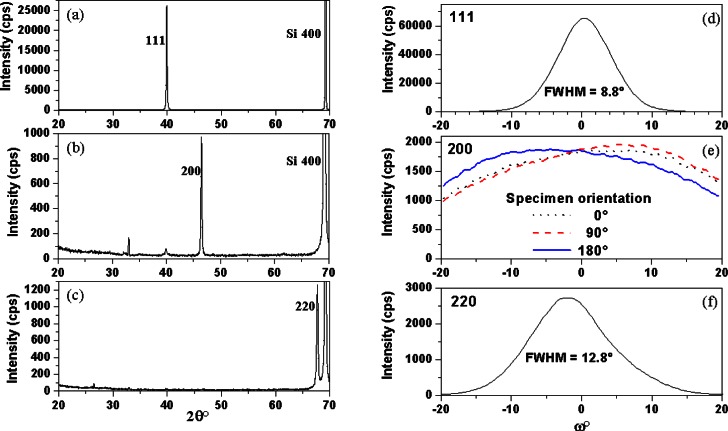
XRD data from Pt (150 nm) / SiO_2_ (300 nm) / Si specimens prepared by dc sputtering with different Ar/O_2_ additions, annealed to cause grain growth and give (111), (100), and (110) texture. Specimens provided courtesy of C. S. Hwang, currently at Seoul National University, Seoul, Korea.

**Fig. 2:**
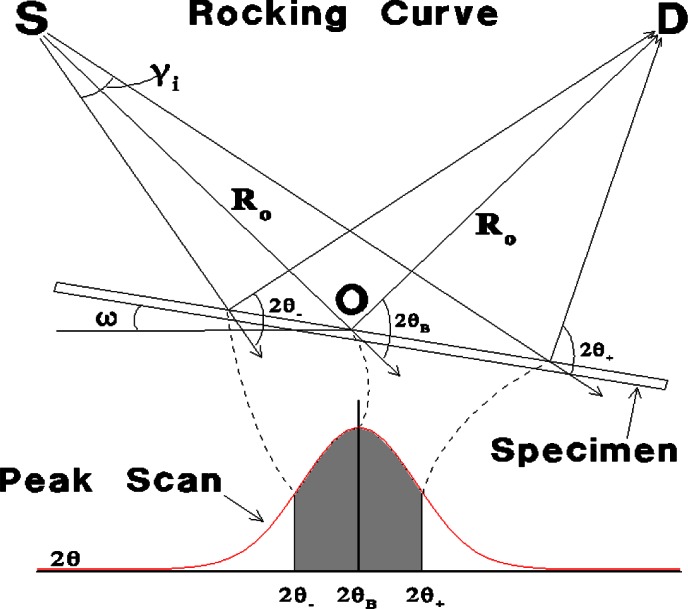
Schematic of x-ray source, specimen and detector showing relation between defocus during *θ* scan and peak scan intensity

Many thin film and bulk materials used in electronic applications have a preferred crystallographic orientation, or texture, and the properties of these materials can be strongly affected by texture. Some of the more important ways in which texture influences materials behavior and properties include: the link between crystal structure and crystal morphology and the use of crystal morphology to create texture; the effect of texture on tensor properties of bulk and thin film ceramics and metals, e.g., piezoelectric constants, elastic coefficients; and the effect of texture on the intensities of diffraction peaks. It is necessary to quantify the effects of texture on properties in order to optimize the development and application of textured materials; clearly, this requires accurate characterization tools. Typically, experimentalists make their initial estimates of texture from the relative intensities of Bragg peaks in conventional *θ*-2*θ* x-ray diffraction (XRD) scans, comparing peak intensities with those obtained from untextured (random) specimens. A peak that is stronger than all the other peaks in a pattern, relative to peaks from a random specimen, identifies a direction of preferred crystallographic orientation that is normal to the diffracting planes for that peak. However, peak intensities in *θ*-2*θ* XRD scans only provide information on diffracting planes that are oriented within a very small angle (half the incident beam divergence and typically ≈0.5°) of the sample surface, and thus the relative peak intensities do not provide a reliable guide to the strength of the texture, as the following example demonstrates. Platinum films were prepared by dc sputtering with controlled Ar/O2 atmospheres, followed by annealing. The conventional XRD patterns in Figs. 1 (a), (b) and (c) show strong 111, 200 and 220 peaks, respectively, collected from three films with different treatments; these spectra also contain the Si 400 peak from the substrate. However the degree of texture achieved in these three films was markedly different as shown in Figs. 1(d)–(f), which are corrected *ω* scans from the three films and give the pole density profile, or texture profile. *ω* is the angular deviation of the specimen plane from the symmetric position, as illustrated in Fig. 2. The *ω* scans provide orientation information over a much wider angular range than the 0.5° of the Bragg peaks discussed above. They will be described in greater detail in Sec. 2. For now, the *ω* scans show that the (111) texture was strongest, with a full width at half maximum (FWHM) of 8.8° and the (110) texture was also fairly strong with a FWHM of 12.8°. However, the (100) texture was weak with a FWHM exceeding 30°. Fig. 1(e) shows corrected *ω* scans for the (100) textured specimen with the specimen placed at three different angles of rotation about the specimen normal. These scans show that the texture, as well as being weak, is asymmetrical. Also, as expected, the 0° and 180° curves are related by a 2-fold rotation about the line *ω*= 0°. The quantitative FWHM results are qualitatively suggested by close examination of the *θ*-2*θ* scans. In Fig. 1(a), the only peak from the Pt film is 111, but in Fig. 1(b), the Pt 200 peak is strong but the 111 peak is also readily apparent, and the 111 Pt peak in Fig. 1(c) is extremely weak but visible nonetheless.

## 2. Development of New Texture Measurement Technique

**Fig. 3:**
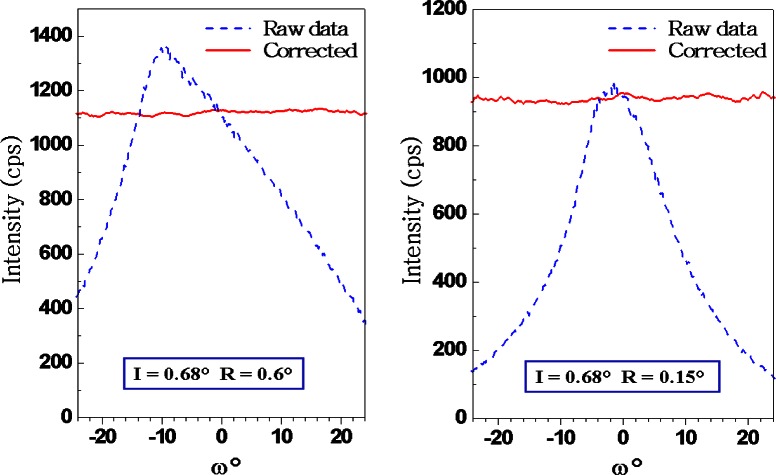
Raw and corrected rocking curves from untextured alumina powder (SRM 676) using 3030 peak with different receiving slits; note that raw data is peaked, corrected data is flat.

The impetus to develop improved texture measurement tools in the Ceramics Division at NIST came in 1994 from American Superconductor Corporation (ASC),^1^ in Westborough, MA. During product development, ASC needed to do rapid, accurate crystallographic texture measurements on wires (or “tapes”) composed of the high temperature superconductor, Bi_2_Sr_2_Ca_2_Co_3_O_7_, swaged in silver. Their goal was to correlate the observed texture with measured electrical and mechanical properties, which are known to be strongly influenced by texture. A major requirement was that the texture measurement technique could be performed using the conventional x-ray powder diffractometer available at ASC at that time. In response to this need, a technique was developed at NIST for quantitative measurement of texture using scans performed on a conventional 2-circle diffractometer [1]. In a similar way to the procedure employed in many other texture analysis methods, an *ω* scan from the textured specimen is divided by an *ω* scan from an untextured sample of the same material to give the texture profile in “multiples of a random distribution” (MRD). The innovative aspect of the new data analysis method is that the *hkl ω* scan from an untextured sample of the same material is calculated, not measured, thus removing the need for the untextured sample, which is frequently difficult to obtain. The input data to the *ω* scan calculation are the optics of the diffractometer and a *θ*-2*θ* scan of the *hkl* peak taken from the textured sample. The calculation takes account of the following: purely geometrical factors such as the variation in irradiated area with incident beam angle and the effect of absorption; and the effect of defocussing, which is the variation in scattering angle at different parts of the irradiated specimen surface when the specimen is tilted out of the symmetrical orientation. This defocussing is shown schematically in Fig. 2, which illustrates how the intensity detected from different parts of the tilted specimen is obtained from the peak scan. Thus the random intensity at a particular *ω* is proportional to an integral over the Bragg peak scan, the integration limits (2*θ*_−_ and 2*θ*_+_) being functions of *ω* and 2*θ*_B_. The experimental scan from the textured sample is divided by the calculated *ω* scan from a random specimen to give the texture profile of the sample. Since the technique employs a two circle diffractometer and is limited to an angular range in *ω* of −*θ*_B_ to *θ*_B_, it is best suited to the analysis of relatively sharp axisymmetric (fiber) texture, although the selection of high Bragg angle peaks allows relatively large orientation ranges to be probed. The software to perform the calculations of the analysis was developed at NIST and successfully transferred to ASC in 1995. The software has since been developed into a Windows-based package called TexturePlus, which is available on the World Wide Web [2]. The technology was validated at NIST using SRM 676, an alumina powder consisting of equiaxed particles that do not texture when pressed into a powder bed for diffraction. Results from these experiments, which include raw and corrected omega scans from SRM 676 specimens, are shown in Fig. 3. The scans were carried out with an incident slit width of 0.68° and two different final slits, 0.15° and 0.6° as indicated. Despite the large deviations from a constant intensity displayed by the raw data, the corrected curves are of constant intensity over the whole range scanned. These collective results demonstrate that omega scans on 2-circle machines with divergent x-ray sources can be used as quantitative tools.

## 3. Applications of the Technique

It became clear that there were many potential users of this method in industry, particularly small businesses, and academia, where more sophisticated texture measurement equipment, such as 4-circle diffractometers and area detectors, is not readily available. Since most advanced electronic materials are used in thin film form, the technique was extended to analysis of diffraction data from thin films. In this case, the thickness and linear x-ray absorption coefficient of the film were necessary input data, and the correction factors were different from those calculated for bulk materials particularly when films as thin as a few tens of nanometers were analyzed.

**Fig. 4:**
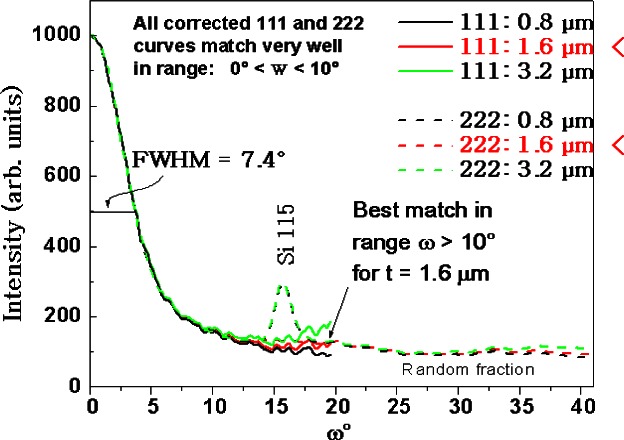
(111) texture measurements from 1.6 μm thick electrodeposited copper films, measured with 111 and 222 peaks and corrected for thicknesses of 0.8 μm, 1.6 μm, and 3.2 μm. Specimens provided courtesy of Semitool Corporation.

Validation of the thin film correction algorithms was achieved in 1998 with data obtained from electrodeposited films of copper, which are replacing physical vapor deposited aluminum films in the new generation of chip interconnection technology. In this study, the (111) texture of 1.6 μm thick electrodeposited Cu films was investigated with two *ω* scans, using 111 and 222 Bragg peaks [3]. Figure 4 shows that the agreement between the 111 and 222 texture profiles is excellent and that when the correction algorithms in TexturePlus are applied using the correct film thickness (1.6 μm) the agreement between the 111 and 222 curves in the region of *ω* = 20° is the best. Also noted is a large randomly oriented fraction. Random fractions give rise to a constant and non-zero corrected intensity for *ω* values above a certain angle which is determined by the sharpness of texture in the textured fraction and in this case is about 15°.

The above copper study is an example of a common phenomenon in which a material, particularly in thin film form, contains two or more differently textured populations. The analysis of bimodal or multimodal texture is an important topic in the characterization of thin film texture [4]. The volume fraction of material in a particular textured population is characterized by joining together the results of two diffraction measurements, the first being the intensity of a Bragg peak diffracted by the textured planes, and the second being the width of the texture profile measured using that peak. The Bragg peak integrated intensities are corrected for the thickness of the film; relative to bulk, thin films diffract less intensity, and this effect is greater at higher incidence angles. The integrated intensity of each *hkl* peak (after thickness correction) is compared with the integrated *hkl* peak intensity from an untextured powder specimen of the same composition (either determined experimentally or obtained from literature sources); this gives the relative abundances of material with the exact *hkl* orientation, E*_hkl_*, within the divergence of the incident beam. A textured volume fraction occupies more of orientation space than is sampled by this divergence. To find the total *hkl*-oriented volume fraction of material, *T_hkl_*, we calculate *X_hkl_*, the integral over all orientation space of the corrected *hkl* rocking curve scaled to a maximum value of one, and multiply it by *E_hkl_*. In experiments on PbZr_1−_*_x_*Ti*_x_* O_3_ (PZT) thin films for non-volatile random access memory, the *X_hkl_* values were similar and the {100} volume fraction was much smaller than the {111} fraction; in this case, the agreement between *E_hkl_* and *T_hkl_* for the {100} and {111} volume fractions was relatively good [4]. In situations where the differently textured volume fractions are similar in size, small differences in the FWHM of the corrected *ω* scans produce significant differences in *X_hk_*_l_ and hence in *T_hkl_*. For an example of this, see Fig. 3 in Ref. [3]; further analysis of the data from the Ba_0.7_Sr_0.3_TiO_3_ (BST) thin films presented in this paper shows that the {110} volume fraction based on peak integrals is underestimated by 50 % compared with the full volume fraction analysis presented above. We also note that in a specimen with a significant random fraction, the random fraction cannot be determined with any accuracy solely from peak integral ratios because *X*_rand_ is by definition one and is therefore typically more than three orders of magnitude larger than *X*_200_ and *X*_222_. This work has shown that in the general case, quantification of volume fractions of textured material requires analysis of the orientation distribution in each textured volume fraction, especially when the degree of preferred orientation is different in the textured volume fractions, or the differently oriented volume fractions are similar in size.

**Fig. 5:**
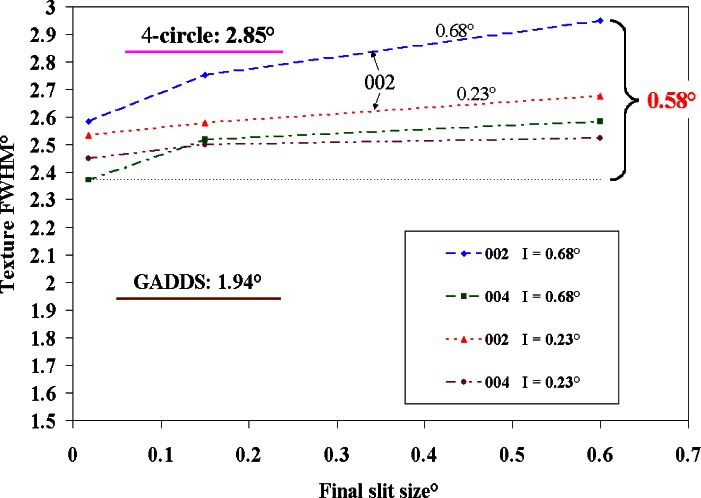
Texture results from ZnO film processed at 650 °C. Powder diffractometer results are plotted as function of final slit size for various incident slit sizes (I). The 4-circle and area detector results are shown as horizontal lines.

A particularly interesting application arose in the case of zinc oxide thin films used for high frequency acoustic resonators in cellular phones under investigation by Agere Systems. The property of importance is the electromechanical coupling coefficient of the films, which is a function of the processing temperature. The films are 750 nm of ZnO on Pt/Si substrates. Processing temperatures have a strong effect on the degree of basal plane texture (ZnO has a hexagonal crystal structure); therefore accurate monitoring of this texture would constitute a valuable diagnostic for film quality assessment. Various tools have been used for this measurement: 2- and 4-circle XRD diffractometers and an area detector diffractometer. The FWHM values of the texture profile, determined by the 2-circle technique using TexturePlus, are plotted in Fig. 5 for various incident slit sizes as a function of receiving slit size; also shown are the 4 circle and area detector results. The results are not consistent, and indicate that not only do different techniques give significantly different results (1.2° difference between 4-circle and area detector) but also that the results from a single method depend on the optics of the x-ray equipment (variation of 0.58° for the 2-circle method). Such discrepancies must be understood and resolved before developing standardized texture measurement methods. The sensitivity to x-ray optics is most important when the texture being measured is sharp, as just described. When a rocking curve was obtained from a perfectly textured material (i.e., a single crystal), it was shown both experimentally and theoretically that the texture profile FWHM was equal to the divergence of the incident slit [5]. The results presented in Fig. 3 show that for weaker textures the choice of receiving slit does not affect the corrected texture profile to any extent.

This paper has shown that there are a number of important issues in texture measurement that remain unresolved. These include the continued use of *θ*-2*θ* scans for texture measurement and the many possibilities for the misinterpretation of these scans, and the lack of agreement between different laboratories when measuring the texture of the same specimen. To address these issues, a workshop on Texture in Electronic Applications was held at NIST Gaithersburg October 10–11, 2000, and was attended by about 40 researchers evenly divided among industry, academia and national laboratories [6]. The primary goal of the workshop was to provide a forum for the discussion of critical issues relevant to texture and texture measurement. At this meeting, the need for a texture standard was voiced by several participants; it was determined that a round robin of measurements on identical specimens using various techniques was the first step in developing a standard. The following institutions have agreed to participate: Oak Ridge National Labs, IBM, HKL Technologies and McGill University; others will be solicited. NIST will coordinate this interlaboratory activity using Pt thin films with axisymmetric preferred crystallographic texture on Si substrates (courtesy of Ramtron Corporation International) as the candidate texture standard.

## 4. Conclusions

Initial estimates of texture are typically made by observing its effect on peak intensities in x-ray diffractograms. NIST has developed tools for more accurate texture measurement, tools that still use the conventional powder diffractometer. Texture is measured by combining a *θ*-2*θ* scan of a peak from the textured planes with an *ω* scan using that peak. The software required to analyze the x-ray data is available on the Web. Bulk and thin film specimens can be analyzed; for thin films the film thickness and linear x-ray absorption coefficient of the material are required. The technique has been applied to many different materials and examples are given of application to ceramics and metals in both thin film and bulk form. A workshop on Texture in Electronic Applications was held at NIST Gaithersburg in the fall of 2000 and there was significant discussion of the need for a texture standard.

## Footnotes

1 Certain commercial equipment, instruments, or materials are identified in this paper to foster understanding. Such identification does not imply recommendation or endorsement by the National Institute of Standards and Technology, nor does it imply that the materials or equipment identified are necessarily the best available for the purpose.
